# Identifying Gaps and Opportunities to Improve Ototoxicity Management in Veterans With Cancer: Evidence From a Retrospective Cohort and Oncology Provider Survey

**DOI:** 10.1002/cam4.71566

**Published:** 2026-02-15

**Authors:** Cecilia Lacey, Khaya Clark, James Riley Debacker, Hunter Stuehm, Michelle Hungerford, Trisha Milnes, Kirsten Johansson, Rosemarie Mannino, Julie N. Graff, Dawn Konrad‐Martin

**Affiliations:** ^1^ VA Pittsburgh Geriatric Research, Education, and Clinical Center Pittsburgh Pennsylvania USA; ^2^ VA Rehabilitation Research, Development & Translation Services Center, National Center for Rehabilitative Auditory Research VA Portland Healthcare System Portland Oregon USA; ^3^ Department of Medical Informatics and Clinical Epidemiology Oregon Health & Science University Portland Oregon USA; ^4^ VA Health Services Research & Development Center of Innovation, Center to Improve Veteran Involvement in Care VA Portland Healthcare System Portland Oregon USA; ^5^ Department of Otolaryngology/Head & Neck Surgery Oregon Health & Science University Portland Oregon USA; ^6^ Department of Audiology and Speech Pathology VA Augusta Healthcare System Augusta Georgia USA; ^7^ Department of Hematology/Oncology VA Portland Healthcare System Portland Oregon USA

**Keywords:** audiology, chemotherapy, drug‐related side effects and adverse reactions, Otology/Neurotology, ototoxicity, professional practice gaps, sensorineural hearing loss

## Abstract

**Purpose:**

Identify factors influencing audiological care for chemotherapy‐induced ototoxicity from the perspectives of oncology providers in the Veterans Health Administration (VA), and quantify audiology service use among Veterans receiving ototoxic chemotherapies.

**Methods:**

We surveyed VA oncology providers to identify barriers and facilitators to ototoxicity management (OtoM). We also conducted a VA‐wide retrospective cohort analysis over a 5‐year period to quantify audiology service use among Veterans who received cisplatin, carboplatin, or oxaliplatin chemotherapy.

**Results:**

A total of 30,643 Veterans received platinum‐based chemotherapy from 2014 to 2019. Few of them (< 10% on cisplatin, < 5% on carboplatin or oxaliplatin) accessed audiology services within a year of treatment. Of the 8702 patients on cisplatin, only 9.6% had two or more audiology encounters. Thirty‐six oncology providers completed our survey. Most providers believed OtoM should be routine for patients on cisplatin (97%) or carboplatin (70%), but they overestimated audiology service provision levels relative to our analysis. Most providers would consider giving a different chemotherapy drug (73%) or decrease the dose (56%) for patients with ototoxicity, yet only 36% routinely referred patients to audiology. Access, perceived need, and resources were major barriers to OtoM, while care coordination was a primary facilitator.

**Conclusions:**

OtoM is a care‐gap for Veterans with cancer, despite its perceived value to VA oncology providers. Cisplatin and carboplatin frequently add hearing loss and tinnitus to survivors' treatment burdens. This study offers insights into oncology providers' views on OtoM, guiding efforts to address the identified care gap.

## 1 Introduction

Patient survival is a primary focus of oncology practice, which is evolving to include closer surveillance of the patient's physical and psychosocial functions. This allows the care team to identify adverse effects of treatment that could necessitate modifications to the treatment plan [1, 2]. Cisplatin‐containing chemotherapy is used in 10%–20% of all cancer patients [3] due to its unrivaled effectiveness in treating solid or central nervous system tumors [4]. Unfortunately, cisplatin treatment also causes permanent damage to the inner ear, affecting both hearing and balance [5, 6, 7, 8, 9]. A recent meta‐analysis indicates that hearing loss after cisplatin chemotherapy occurs in 49.21% of patients (CI 42.62%–55.82%) when pooled across tumor types and age groups [5]. Among Veterans, the incidence of cisplatin‐related hearing shifts ranges from 46% to 76% [6], with nearly 40% acquiring new tinnitus [7]. The vestibulotoxic effects of cisplatin are less well studied, and vestibular function is not routinely measured by audiologists when evaluating ototoxicity [8, 9]. Ototoxicity‐related hearing loss, tinnitus, and imbalance have been linked to reduced physical and psychosocial function and poorer quality of life among cancer survivors [10, 11, 12, 13]. Because cisplatin is often reserved for patients with curable cancers, these individuals may endure the consequences for decades [1, 5, 11, 12, 14]. This study aims to quantify the care gap in ototoxicity management (OtoM) in the VA setting and offers insights into oncology providers' views on addressing this gap.

Patients and providers consistently underestimate the severity of treatment‐related ototoxicity [14, 15]. This highlights the importance of proactive symptom surveillance and management to prevent further damage and address the functional loss through rehabilitative interventions. These goals correspond to “primary prevention” and “secondary prevention” in the parlance of public health frameworks [16, 17] and are the main clinical objectives for ototoxicity monitoring per the American Academy of Audiology [18], American Academy of Otolaryngology‐Head and Neck Surgery [19], American Speech‐Language Hearing Association [20], International Ototoxicity Management Group [21], and World Health Organization [22]. Although some guidelines refer specifically to “ototoxicity monitoring” we use the broader term “ototoxicity management (OtoM)” [21] throughout this manuscript to emphasize the importance of both symptom surveillance and responsive clinical action.

Recommendations for OtoM initially focused on preventing hearing loss but have expanded to include functional measures for speech understanding in noise, as well as tinnitus and balance problems [23]. Educating and counseling patients and their oncology teams is key, helping them to utilize the information when ototoxicity is identified [18, 19, 20, 21, 22]. Effective OtoM should create opportunities for oncologists to modify treatments when appropriate, and for audiologists to engage the patient in a program of hearing, tinnitus, and/or balance rehabilitation, reducing loss to follow up [18, 19, 20]. Although the impacts of OtoM interventions on functional status are not fully described [23], many patients with cancer value preserving their pre‐treatment function nearly as much as they value their survival [24].

Despite strong support from medical and health associations, OtoM is not routinely provided for adults with cancer [25, 26]. Audiology services for managing ototoxicity are often disconnected from cancer care and underutilized [27, 28, 29, 30, 31, 32]. This care gap persists despite evidence showing that rehabilitation for auditory dysfunction can improve functional outcomes in healthy older adults [33] and mitigate certain impairments during and after oncology treatment [34]. In 2006, the National Cancer Institute recommended integrating rehabilitation services into the oncology care continuum and as an essential component of survivorship care [35]. Although progress has been made since this initial report, subsequent updates reveal remaining gaps in care to identify and treat functional loss, and encourage using “implementation science strategies to translate findings [on effective interventions] into practice” [17, 36].

Interventions that are deemed efficacious in research studies often fail to be implemented in real‐world settings [37] because healthcare systems are highly complex [38], and barriers to intervention implementation occur at multiple levels [39, 40]. The Consolidated Framework for Implementation Research (CFIR) is a conceptual framework designed to guide the intervention design and implementation [41, 42]. CFIR focuses on five domains (i.e., intervention characteristics, inner setting, outer setting, process of implementation, characteristics of individuals). Within the Veterans Health Administration (VA), CFIR has been used to support the implementation of interventions such as Whole Health System transformation, opioid risk tools, and harm reduction services by identifying barriers and facilitators to adoption [43, 44, 45]. Consistent with this body of work, it will be necessary to thoughtfully consider clinician and patient stakeholder perspectives to successfully implement programmatic OtoM in the VA.

Using CFIR, we previously developed and used the Ototoxicity Management Interdisciplinary Care (OtoMIC) Survey to assess VA audiologists' perspectives on OtoM [29]. Findings highlighted the need for clearer interdisciplinary communication between audiologists and oncology teams, and clearly defined provider roles for OtoM [30]. The present report is a companion study that uses the OtoMIC survey to characterize VA oncology providers' perspectives on OtoM. We also report on a VA‐wide retrospective cohort analysis to quantify audiology service use among Veterans who received cisplatin, carboplatin, or oxaliplatin chemotherapy, treatments which varies in their ototoxic effects from most to least [18].

## 2 Methods

### 2.1 Survey Development and Validation

Details on the OtoMIC survey development are described elsewhere [29]. The 26‐item, anonymous survey and its analysis are informed by three CFIR constructs: characteristics of individuals (providers' experience, knowledge, and beliefs regarding OtoM); outer setting (patients' OtoM needs and organizational prioritization for addressing these needs); and inner setting (structural characteristics of the healthcare system or facility). We used the inner setting's implementation climate domain to assess barriers and facilitators through open‐ended questions. Branching logic was employed to slightly alter questions based on whether the respondent was an audiologist or oncology provider, and whether they were a clinician or service chief.

Survey face validity was established through iterative review by a multidisciplinary panel of subject matter experts and an implementation science/health service delivery expert to ensure that questions captured important dimensions of OtoM and aligned with the conceptual framework. The oncology version of the OtoMIC survey is provided in Appendix A: Table A1 and can be used or adapted with permission from the authors. The full survey is available upon request. Appendix A: Figure A1 displays the relationship between the survey, CFIR constructs, and the long‐term project goal—to guide efforts for integrating OtoM into routine cancer care.

### 2.2 Data Collection

The study PI (DK‐M) first invited oncology providers at the VA Portland Healthcare System to participate in the survey, in order to refine the survey for language clarity and estimate its 15‐min completion time. Initial feedback was also gathered after the PI's VA Extension for Community Healthcare Outcomes (ECHO) presentation [46]. Subsequently, study team member (CL) emailed 93 VA oncology team members (oncologists, oncology nurses, and pharmacists) using addresses from national VA listservs as per IRB specifications. Oncologists at 12 National Cancer Institute and VA Interagency Group to Accelerate Trials Enrollment (NAVIGATE) sites, the VA National TeleOncology Program, and Prostate Cancer Foundation Centers of Excellence were prioritized. Two follow‐up reminder emails were sent. Respondents completed the survey via Qualtrics between June 2022 and October 2023 using Qualtrics, a secure web‐based application.

To complement survey responses and further characterize the scope of OtoM practices in the VA, a historical cohort of 30,643 unique patients was developed for the 5‐year period prior to the pandemic; 2015–2019. Patients who received cisplatin, carboplatin, or oxaliplatin as first‐line treatment were identified from pharmacologic records in the VA Cancer Registry. Individuals whose cancer recurred were counted only once. These data were linked to corresponding audiology records in the VA Corporate Data Warehouse. This cohort included patients who received private audiology care purchased through the VA Community Care program. The percentage of patients who visited audiology at least once between 1 month before and 1 year after their first platinum‐containing treatment was calculated, as well as the per patient number of audiology appointments. Patients receiving care at VA cancer centers conducting large clinical trials, including NAVIGATE sites, were analyzed separately.

### 2.3 Quantitative Analysis

The primary research aim was to understand the current landscape of OtoM from the perspective of oncology providers and barriers/facilitators associated with its provision. Quantitative survey data were analyzed using descriptive statistics and frequencies. Results are reported as absolute frequency (i.e., number of respondents selecting each option), relative frequency (i.e., percentage of responses including a given option), or median (i.e., for nonparametric estimates of central tendency). The number of respondents completing each question is noted, as not every respondent answered every question. Inferential statistics were not performed, as the survey was not designed to test a null hypothesis.

### 2.4 Qualitative Analysis

Qualitative data from the survey were analyzed using inductive thematic analysis [47]. Respondents were asked four open‐ended questions regarding the implementation climate for OtoM (e.g., preferred ototoxicity monitoring schedule, barriers to implementing ototoxicity monitoring at their facility, perspectives on additional changes or resources needed to enhance OtoM, and types of OtoM changes that leadership would support). A final question invited participants to share additional comments or recommendations related to OtoM at their facility.

Two members of the project team (CL and HS) independently reviewed the qualitative responses, developed an initial codebook, and tagged text related to OtoM barriers and facilitators. The team met regularly with the lead analyst (KC) and the study PI (DK‐M) to discuss the data and revise the codebook. This process included three iterative cycles of codebook revision, coding, and analysis. The entire project team met several times to review output files and discuss the interpretation of findings. Reflexivity guided the analysis and interpretation process, which refers to ongoing self‐reflection and acknowledgment of personal biases during analysis [48]. Atlas.ti software [49] was used to support the qualitative analysis and all data were securely stored in compliance with VA policy.

## 3 Results

### 3.1 Demographics

Thirty‐six participants responded to the OtoMIC survey, and 31 completed it in its entirety. Forty‐one percent of respondents were oncologists. Among these oncologists, 19% were Oncology Service Chiefs or Leads. Fifty‐four percent of respondents were nurses, 19% of whom were advanced practice providers. Six percent of respondents were pharmacists.

Most respondents (94%) worked at a VA Medical Center, while 6% worked at both a VA Medical Center and a Community‐Based Outpatient Clinic. Participants represented multiple oncology settings, which varied by cancer type, treatment goal, and service delivery model (e.g., VA National TeleOncology Program). Most respondents (78%) had been practicing in their field for more than 10 years. At least one response was received from 9 of the 12 NAVIGATE sites and 13 of the 18 VA regional systems of care (Appendix B: Figure B1). Three respondents did not provide their specific location.

### 3.2 Characteristics of Individuals: Oncology Providers' OtoM Experience, Knowledge, and Beliefs

**FIGURE 1:**
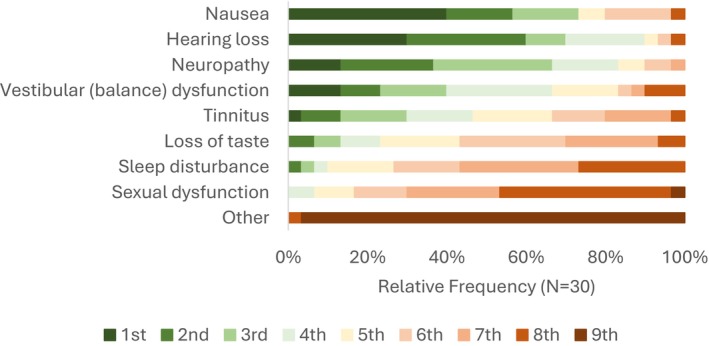
Ranking of non‐lethal side effects of chemotherapy treatment according to the importance of managing each symptom. Participants ranked nine non‐lethal side effects from most important (1st) to least important (9th) to manage. For example, 12 of 30 respondents (40%) ranked nausea as the most important symptom to manage, and 9 of 30 respondents (30%) ranked hearing loss as the most important. (Survey Question 21: “Please rank the importance of managing the following treatment side effects.”)

Questions related to the CFIR characteristics of individuals construct explored oncology providers' perspectives on OtoM. In Survey Question 21, respondents were asked to rank the importance of managing various non‐lethal side effects of chemotherapy. Nausea and hearing loss were ranked as the most and second most important side effects to manage, respectively, ahead of other toxicities such as neuropathy, vestibular dysfunction, tinnitus, loss of taste, sleep disturbance, and sexual dysfunction (Figure 1).

Respondents also ranked the importance of various OtoM program considerations. Feasibility, such as availability of patients, staff, and equipment, and the relevance of test results to the oncology treatment plan were ranked as the first and second most important considerations, followed by factors such as patient health status, patient preference, relevance to rehabilitation planning, and cost (Appendix C: Figure C1).

The majority of respondents (97%) felt that some form of ototoxicity monitoring (symptom surveillance) was needed for patients receiving cisplatin. Monitoring was also perceived as necessary for patients receiving carboplatin (70%), oxaliplatin (67%), and radiation (94%).

Regarding when monitoring should occur (i.e., what they believe is an appropriate schedule for the results to be useful/actionable), “when a patient reports ototoxic effects/symptoms” and “[at the] beginning/end of treatment” were more frequently selected than options such as “prior to administration of each dose” or “after every cycle” (Table 1).

**TABLE 1:** Preferred surveillance schedules for monitoring ototoxic hearing loss, as a function of treatment type.

	Cisplatin (*N* = 34)	Carboplatin (*N* = 33)	Oxaliplatin (*N* = 33)	Radiation (*N* = 34)
When patient reports ototoxic effects	24 (71%)	21 (64%)	21 (64%)	19 (56%)
Beginning and end of treatment	27 (79%)	13 (39%)	10 (30%)	20 (59%)
After every cycle	8 (24%)	7 (21%)	6 (18%)	7 (21%)
No monitoring is needed	1 (3%)	10 (30%)	11 (33%)	2 (6%)
Prior to each dose	6 (18%)	4 (12%)	3 (9%)	3 (9%)
Unsure	1 (3%)	2 (6%)	3 (9%)	8 (24%)

*Note:* Respondents selected all monitoring schedules they believed would provide useful and actionable results for patients receiving cisplatin, carboplatin, oxaliplatin, or radiation. Higher percentages indicate more commonly selected options and are color coded in dark green. Lower percentages indicate less commonly selected options and are color coded in light green, or white. (Survey Question 14: “What do you believe is an appropriate schedule of ototoxicity monitoring tests for the results to be useful/actionable for you and your patients?”).

Respondents also rated the perceived usefulness of different OtoM program elements. Most rated every element as extremely or very useful: management of ototoxic effects during treatment (93%), baseline evaluation (93%), point‐of‐care ototoxicity screening (90%), early detection (87%), management of ototoxic effects after treatment (87%), hearing health education and resources (87%), and the ability to influence treatment planning (78%) (Appendix C: Figure C2).

Finally, respondents were asked about action plans in response to three clinical scenarios involving patients who reported ototoxic symptoms. The scenarios varied by symptom type (e.g., difficulty following conversations, tinnitus, significant hearing shift), chemotherapeutic agent, and impact on patient function and tumor response. Averaged across the scenarios, most oncology providers indicated they would consider adjusting the treatment agent (73%) or dosage (56%) (Table 2). Only two respondents indicated they would never consider changing treatment. Related open‐ended responses provided additional context (see “Barriers and Facilitators Associated with OtoM” section).

**TABLE 2:** Preferences for various action plans in response to specific clinical scenarios involving ototoxicity.

	Scenario 1 (*N* = 35)	Scenario 2 (*N* = 35)	Scenario 3 (*N* = 34)
Refer to audiologist for management	33 (94%)	29 (83%)	22 (65%)
Increase the frequency of ototoxicity monitoring	25 (80%)	17 (71%)	16 (71%)
Consider changing the agent in the next cycle	25 (71%)	20 (57%)	31 (91%)
Provide counseling	20 (57%)	23 (66%)	24 (71%)
Consider changing the dosage in the next cycle	28 (71%)	25 (49%)	23 (47%)
Other	0 (6%)	2 (6%)	1 (6%)
No treatment change	2 (0%)	2 (6%)	2 (3%)

*Note:* Respondents selected all applicable actions for three clinical scenarios describing patient‐reported or audiologically confirmed ototoxicity. Scenarios varied by chemotherapeutic agent, symptom type (e.g., difficulty hearing, tinnitus, significant hearing shift), impact on patient function, and tumor response. Higher percentages indicate more commonly selected actions. (Survey Question 13: Scenario 1: A family member reports the patient is having trouble following conversations in noise after cisplatin. Scenario 2: A patient reports ringing in the ears before a new cycle of carboplatin and radiation. Scenario 3: Audiologist confirms a significant hearing shift after cumulative cisplatin exposure; the patient is concerned about worsening hearing loss despite a good tumor response). Higher percentage in the table indicate more commonly selected options and are color coded in dark green. Lower percentages indicate less commonly selected options and are color coded in light green, or white.

### 3.3 Outer Setting: Patients' OtoM Needs and Organizational Prioritization

Patients' OtoM needs and organizational prioritization were assessed through questions mapped to CFIR's outer setting domain. Oncology providers were asked to approximate the number of patients in their monthly caseload receiving platinum‐based chemotherapy and/or radiotherapy. Estimates varied widely, and many respondents chose not to answer (Appendix C: Table C1).

Respondents estimated the percentage of their patients who experienced new or worsened side effects due to ototoxic agents. While estimates ranged from 0% to 100%, median estimates were 10% for new or increased hearing loss, 10% for tinnitus, 10% for balance changes, and 15% for decreased quality of life (Appendix C: Figure C3). On average, respondents' estimated rates of ototoxic hearing loss for cisplatin and carboplatin were approximately half of what has been reported in the literature [5] (Appendix C: Figure C4).

### 3.4 Inner Setting: Healthcare System Structural Characteristics

Finally, inner setting factors related to healthcare system structure were explored. Most respondents reported that audiology services were commonly provided to patients receiving cisplatin (68%), but less commonly for patients receiving carboplatin (25%), oxaliplatin (26%), or radiation alone (32%) (Table 3).

**TABLE 3:** Reported implementation of audiological monitoring for ototoxicity by treatment type.

	Yes	No	Unsure
Cisplatin (*N* = 28)	19 (68%)	8 (29%)	1 (4%)
Carboplatin (*N* = 28)	7 (25%)	19 (68%)	2 (7%)
Oxaliplatin (*N* = 27)	7 (26%)	17 (63%)	3 (11%)
Radiation (*N* = 28)	9 (32%)	9 (32%)	10 (36%)

*Note:* Respondents indicated whether patients typically received audiological monitoring if treated with cisplatin, carboplatin, oxaliplatin, or radiation. Results are compared with VA‐wide retrospective cohort data in the text. (Survey Question 6: “If [drug] is part of the treatment, do oncology patients typically receive audiological monitoring for ototoxicity?”).

Survey questions about team member responsibilities indicated that oncology providers reported being responsible for many aspects of OtoM, including informing patients about the risks of ototoxicity, monitoring patient‐reported symptoms, counseling patients who develop ototoxicity‐related symptoms, and monitoring hearing during treatment with ototoxic agents (Appendix C: Table C2). Audiologists were viewed as primarily responsible for providing aural rehabilitation, as well as assisting with patient counseling and hearing monitoring. In a related question, most respondents (75%) indicated that education and counseling about the risks and impacts of ototoxicity were within an audiologist's scope of practice (Appendix C: Figure C5). Additionally, 89% of oncology providers reported that they personally counsel patients about the possibility of ototoxic effects when prescribing highly ototoxic agents. Most respondents (84%) reported that patients access OtoM services through an oncology referral at their facility (Appendix C: Table C3), but fewer (36%) indicated that they consistently refer patients to audiology for ototoxicity monitoring (Appendix C: Figure C6).

### 3.5 Barriers and Facilitators Associated With OtoM

Several open‐ended survey questions explored barriers and facilitators related to the implementation of OtoM. A summary of these findings can be found in Table 4. A major barrier identified by respondents was accessing audiological services. Many comments described issues related to limited resources, such as audiology clinic wait times. Another reported barrier was a lack of awareness regarding the need for ordering OtoM. Regarding the lack of perceived need for OtoM, one participant stated, “Just do not think it is needed in most cases” (Participant 23).

**TABLE 4:** Key themes of reported barriers to ototoxicity management implementation from the qualitative analysis.

Theme	CFIR domain	Example quotations
Accessing auditory testing	Inner setting	“Time to start treatment vs. time to get into audiology” (Participant 4) “Availability of testing/audiologist” (Participant 24) “Obtaining audiology appts with short notice for patients initiating chemo” (Participant 34)
Perceived need for OtoM	Inner setting	“MD doesn't order” (Participant 4) “Lack of perceived need” (Participant 11) “Just do not think it is needed in most cases” (Participant 23)
Lack of resources	Inner setting	“Time constraints” (Participant 2) “Staffing, limited clinic time, and limited clinic space” (Participant 33) “Staffing at some VAs” (Participant 27)
Managing care coordination	Inner setting	“Audiologist who screens infusion patients for need for evaluation” (Participant 11) “Dedicated cancer care coordination nursing to ensure comprehensive services are automatically integrated (Participant 17)” “Would like all ototoxic agents to require audiology referral before starting” (Participant 4)

*Note:* Themes identified from inductive thematic analysis of open‐ended responses are listed along with their corresponding CFIR domain (inner setting) and selected example quotations from participants.

A key facilitator associated with OtoM implementation was improved care coordination. Participants indicated that enhanced communication and collaboration among oncology, audiology, and other specialties could help better manage and coordinate patient care. Several comments emphasized the need for dedicated staff to facilitate delivery of comprehensive services such as audiological screening, referrals, and rehabilitation. For example, one participant briefly stated, “Just need audiology staff as well as oncology nurse navigator” (Participant 7). Notably, all of the reported barriers identified through the open‐ended responses mapped to the CFIR inner setting construct, the internal environment in which the implementation is taking place.

### 3.6 VA‐Wide Retrospective Cohort

Administrative data from the VA Cancer Registry and Corporate Data Warehouse identified 30,643 unique patients who received cisplatin, carboplatin, or oxaliplatin as first‐line treatment across the VA system between 2015 and 2019. Among these patients, only 2336 (7.6%) accessed audiology services at least once between the month before and the year after their initial chemotherapy treatment.

**FIGURE 2:**
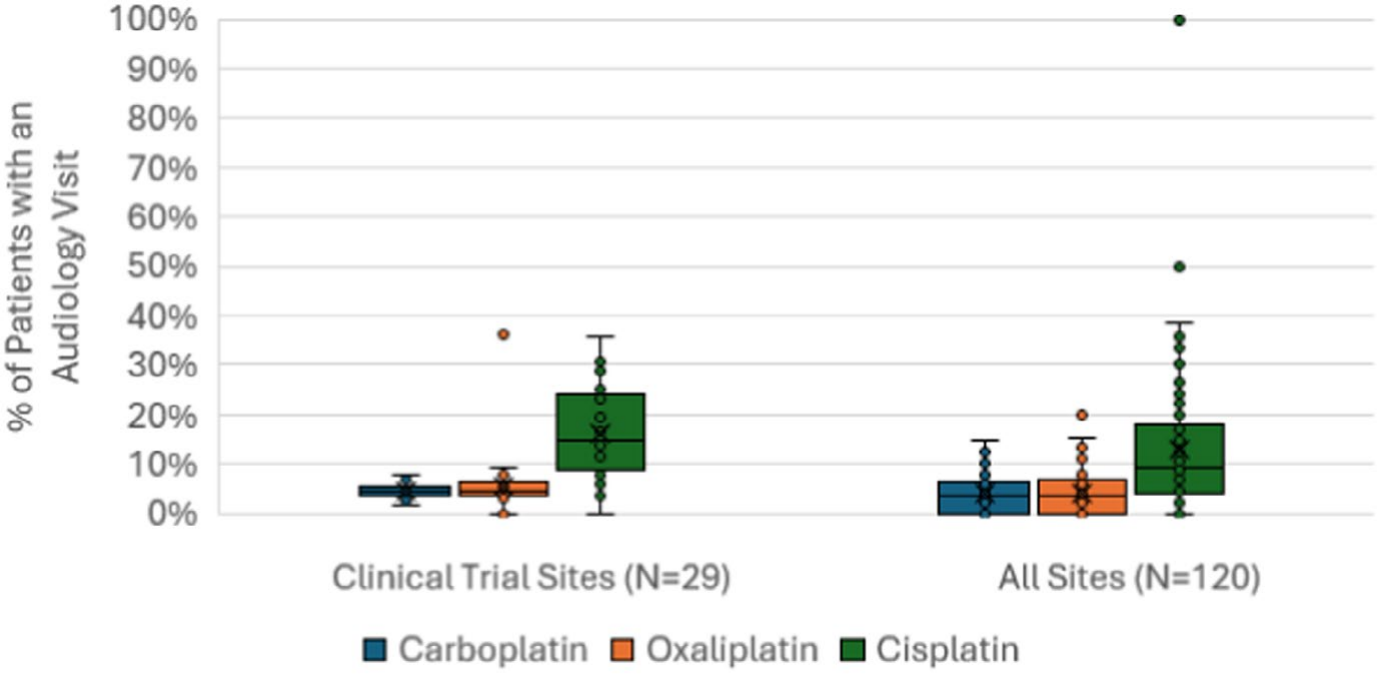
Retrospective cohort study results showing the proportion of Veterans who received an ototoxic chemotherapy agent and accessed audiology services at least once during the surveillance period, by chemotherapy drug. The circles indicate data from each individual VA site, while the box and whisker plots indicate the median and range of values across sites. The figure compares audiology service utilization rates at VA cancer clinical trial or NAVIGATE sites (left) versus all VA sites with qualifying patients (right).

The administrative data in Figure 2 compare audiology service utilization across all VA sites (right) versus VA cancer clinical trial and NAVIGATE sites (left). The circles in Figure 2 indicate the percent of patients seen by audiology at each individual site, while the box and whisker plots indicate the median and range of values across sites. Across all sites (right), fewer than 10% of patients treated with cisplatin accessed audiology services, and fewer than 5% of patients treated with carboplatin or oxaliplatin accessed services. Audiology service utilization was highest for patients receiving cisplatin at centers designated as a clinical trial or NAVIGATE site (left). Even at these sites with presumed high standards of care, on average only 17% of patients receiving cisplatin were seen by audiology, indicating that substantial gaps remain.

This system‐wide retrospective cohort analysis benchmarks the actual care gap in OtoM in VA. However, the error bars for cisplatin reveal high variability between sites for this highly ototoxic drug. A small number of facilities achieved audiology utilization rates reaching half or more of their patients, indicating that site‐level factors also influence OtoM implementation.

In addition, we examined the mean number of audiology visits among the 8702 cohort patients who received cisplatin. Only 843 patients (9.6%) had two or more audiology encounters (range 0–25 visits; median = 0 visits). These findings suggest that few patients received ototoxicity monitoring at American Speech‐Language Hearing Association (ASHA)‐recommended time points, which specify that visits should occur before and after cancer treatment, and at each cisplatin dose [20].

## 4 Discussion

### 4.1 Key Findings

We surveyed oncology providers to identify factors influencing audiological care for Veterans receiving ototoxic cancer treatments. We also conducted a system‐wide retrospective cohort analysis to quantify actual audiology service utilization by Veterans with cancer.

A total of 30,643 Veterans received platinum‐based chemotherapy from 2014 to 2019. Few of them (< 10% on cisplatin, < 5% on carboplatin or oxaliplatin) accessed audiology services within a year of treatment. Of the 8702 patients on cisplatin, only 9.6% had two or more audiology encounters. Thirty‐six oncology providers completed our survey, representing 13 of the 18 VA regional systems. Most providers believed OtoM should be routine for patients on cisplatin (97%) or carboplatin (70%), but they overestimated audiology service provision levels relative to our analysis. Most providers would modify the chemotherapy agent (73%) or dose (56%) for patients with ototoxicity, yet only 36% routinely referred patients to audiology. Access, perceived need, and resources were major barriers to OtoM, while care coordination was a primary facilitator.

Given the VA's role as the largest integrated healthcare system providing cancer care in the United States, these findings highlight an important opportunity for system‐wide quality improvement. Further efforts to strengthen OtoM in the VA, using input from VA clinicians, could also serve as a model for broader improvements in cancer‐related audiology care, particularly since care gaps have been identified in many other healthcare settings.

### 4.2 Previous Retrospective Cohort Studies on OtoM

A few retrospective cohort studies have investigated audiology encounters for patients treated with ototoxic chemotherapies, limited to cancer centers within comparatively small healthcare systems [26, 50, 51, 52]. Lester et al. [51] reported that in a cohort of 149 patients treated with cisplatin at a tertiary hospital in Brisbane, Australia, 56% of patients visited audiology, which is substantially higher than the rate reported here for VA. However, the frequency of audiology encounters for each patient was similarly low: the median number of audiology visits per patient was 0, interquartile range 0–1. In the Australian study, audiology encounters tended to occur early in cancer treatment. This is consistent with a separate single‐institution retrospective cohort study conducted at the Washington University School of Medicine in Saint Louis, Missouri, USA, in which Lee et al. [52] provided the rates of audiograms at various longitudinal time points among adults who received cisplatin‐based chemoradiation for treatment of head and neck cancer. They found that among the 294 patients in the cohort, 220 (75%) had at least one audiogram, but only 58 patients (20%) had more than one audiogram. Most patients were evaluated within 6 months following their initial cisplatin treatment (*N* = 203, 92%). Additionally, of the 156 patients prescribed a hearing aid, only 39 individuals (25%) obtained one, indicating a low rate of hearing treatment adoption. This highlights the importance of systems to actively prevent loss to follow‐up [30]. Both studies reported higher utilization rates of audiology services than the VA. While the reasons for this discrepancy are unclear, the authors noted that the proximity of clinical departments may have supported greater awareness of audiology services and easier coordination of appointments.

### 4.3 Survey Studies on OtoM

The CFIR is a practical framework that helps guide the systematic assessment of potential barriers and facilitators for a healthcare intervention, providing knowledge to tailor strategies that can improve implementation. We previously used the CFIR to develop the OtoMIC survey and to gain the perspectives of VA audiologists about ototoxicity and OtoM in VA [29]. Here we use the OtoMIC survey to better understand the perspectives of VA oncology providers.

CFIR's characteristics of individuals construct states that an intervention's success is influenced by providers' belief in its value [40]. According to our survey results, VA oncology providers highly value most aspects of OtoM, and the majority reported they would consider adjusting treatment if an ototoxic effect occurred. Nearly half indicated that ototoxicity significantly impacts patients' quality of life. Despite recognizing the importance of OtoM, the oncology providers surveyed indicated that they do not routinely refer patients to audiology, even though a high percentage (84%) indicated that referral from oncology is the main pathway for patients to access OtoM within the VA. Thus, our survey results suggest that limited referrals may contribute to the low actual rates of OtoM observed in VA administrative data.

It is also important to note that a small number of oncology providers in our survey felt that OtoM was not necessary for their patient population. Open‐ended responses suggested that variations in the perceived importance of OtoM were based on cancer type, treatment goals, and service‐delivery models. Several providers emphasized that treatment adjustment decisions are multifactorial, suggesting the need for a flexible, individualized approach to OtoM. This is consistent with the approach outlined by the International Ototoxicity Management Group in their 2024 Roadmap to a Global Template for Implementation of Ototoxicity Management for Cancer Treatment [23].

According to CFIR's outer setting construct, it is essential to establish that there is a crucial need for an intervention from the perspectives of all stakeholders (i.e., patient, provider, institution). The oncology providers surveyed reported a lower prevalence of ototoxic hearing loss among patients receiving cisplatin than described in the literature [5]. Similarly, audiologists in the companion study underestimated ototoxicity prevalence [29]. This underestimation of ototoxicity highlights an educational opportunity, which could be addressed through system‐wide initiatives such as the Cancer Rehabilitation VA Extension for Community Healthcare Outcomes (ECHO) Program [46].

Surveyed oncology providers reported being responsible for multiple aspects of OtoM, including counseling, monitoring symptoms, and discussing risks, while audiologists were viewed mainly as responsible for rehabilitation and ongoing monitoring after treatment had ended. The companion study [29] revealed some differences of opinion by the audiology respondents to the OtoMIC survey. Audiologists felt they were responsible for monitoring hearing during treatment. There was also ambiguity among audiologists about role delineation for certain aspects of OtoM. Some audiologists indicated that they were “unsure” which provider was specifically assigned to key tasks, such as communicating the results of ototoxicity monitoring to patients or monitoring patient‐reported ototoxicity symptoms, while other audiologists indicated that “no provider” was assigned or that multiple providers were. CFIR's inner setting construct emphasizes the importance of understanding provider perspectives on role delineation when designing intervention protocols. These results suggest that interdisciplinary discussion between audiologists and oncologists, and perhaps formalized agreements, are needed to clarify roles and improve collaboration around OtoM practices.

Thematic analysis of open‐ended responses also identified that accessing audiology services is a major barrier to OtoM implementation. A small clinical trial at the VA Portland Health Care System found that patients value hearing healthcare during cancer treatment but encounter logistical barriers, and that offering hearing screening directly in oncology infusion units mitigates some of these obstacles [6]. Specific barriers reported in the present study included a lack of resources (e.g., time, space, and equipment) for audiological testing and skepticism about OtoM's impact on cancer treatment outcomes. As Participant 31 noted, there is “inadequate data regarding effectiveness of [OtoM] interventions and impact on cancer treatment outcome.” While a growing body of literature demonstrates links between development of ototoxicity and increasing functional loss among cancer survivors [12, 13], there is a dearth of data on the extent to which OtoM serves as a mediator of functional disability [23, 30], emphasizing the need for research in this area. While 94% of oncology providers surveyed believed OtoM should be provided to patients receiving cisplatin, the most common response when asked what they believed is an appropriate ototoxicity monitoring schedule for results to be useful/actionable was “When a patient reports ototoxic effects/symptoms” and “[at the] beginning/end of treatment.” Audiologists surveyed in our companion paper had varied opinions on the most useful/actionable schedule for patients receiving cisplatin [29]. Overall, our findings align with previous survey results that have identified barriers such as lack of interdisciplinary communication, unclear referral pathways, limited provider knowledge, and absence of standardized protocols [6, 13, 26, 30, 53, 54, 55, 56, 57, 58, 59, 60, 61, 62]. Most of these barriers align with the inner setting within the CFIR, which refers to the internal context of the organization where the implementation is taking place. This is helpful to know given that high‐reliability organizations, such as the VA, strive to promote a culture of agency for making needed changes with the goal of providing the highest quality care.

A recent survey of 120 oncologists at the University of Pittsburgh's Hillman Cancer Center by Chattaraj et al. [50] revealed that monitoring cisplatin‐induced ototoxicity is not routinely completed there. Most (97%) of the oncologists reported that they discuss the risk of ototoxicity with all patients before receiving cisplatin, and many (65%) felt that ototoxicity would impact cancer treatment decision‐making. However, only 18% of the respondents reported that they routinely obtain audiograms for their patients before administering cisplatin, and 35% of the respondents said they do not perform regular monitoring for ototoxicity during treatment. Nearly half of the respondents (47%) also reported that ototoxicity significantly impacts their patients' quality of life, indicating that these oncology providers perceive a need for routine monitoring and prevention strategies. Interestingly, respondents were highly receptive to adopting any OtoM strategies shown to improve outcomes for patients, again indicating the need for high‐quality evidence of impact.

Thematic analysis in the present survey study identified that facilitators of OtoM implementation include care coordination between oncology and audiology services. Respondents emphasized the importance of assigning dedicated staff, such as oncology nurse navigators, to assist patients and families in navigating services, thus improving continuity of care [63]. Previous studies support the idea that service‐delivery models emphasizing patient convenience, such as flexible audiology scheduling [60], hearing screenings based in chemotherapy infusion centers [6], and tele‐audiology [64], can enhance OtoM feasibility.

### 4.4 Clinical Implications

The use of highly ototoxic chemotherapeutics is a mainstay of cancer treatment and ototoxicity is unavoidable in some cases. VA oncology and audiology providers think that OtoM should become an integral part of comprehensive cancer care to help address ototoxicity in their patients. The term “ototoxicity management” (OtoM) [21] rather than “ototoxicity monitoring” invokes the need for patients and their providers to act on the information obtained by audiologists to reduce the long‐term impacts of hearing loss, tinnitus, and balance problems resulting from treatment. Our findings indicate that many oncologists would consider treatment modification to minimize ototoxic exposure (primary prevention) if a patient experiences ototoxicity. However, among the small percentage of Veterans with cancer who accessed audiology services in our retrospective cohort analysis, most did so only once, which is insufficient to enable primary prevention of ototoxicity. Further, few patients receiving ototoxic cancer treatments accessed audiology services at all. This suggests that most patients are not benefitting from secondary prevention of ototoxicity either—which could address auditory or balance function changes through hearing aids and other forms of rehabilitation.

Challenges integrating rehabilitation into cancer care extend beyond OtoM [32, 65]. Yet current standards emphasize comprehensive cancer care, stipulating that this care should identify and address functional loss throughout the cancer care continuum to maintain or restore function, reduce treatment‐related symptom burden, maximize independence, and improve quality of life [32, 65, 66]. Although initiatives are emerging to streamline rehabilitation referrals, audiological services are often excluded from these frameworks [67, 68, 69]. Our survey results provide valuable insights into oncology providers' perspectives on ototoxicity management, which are crucial for addressing the gaps in care highlighted by our administrative data.

### 4.5 Limitations

The retrospective cohort analysis and the oncology providers surveyed in this study were restricted to the VA healthcare system. Although understanding how OtoM integrates into the VA can assist with implementation at different facilities, similar analyses are needed to identify the unique challenges facing other healthcare systems. The OtoMIC survey, provided in Appendix A, can be used to gain site‐specific information.

Additional limitations include a small sample size and the risk of selection bias for the survey portion of this study. The respondents who chose to complete the survey, while based at a wide range of VA clinical trial and NAVIGATE sites and integrated service networks, may be providers that prioritize OtoM. If so, this would result in an underestimation of care gaps. A future study is warranted that could be designed to achieve a larger sample size with a larger number of purposively sampled sites. An additional benefit would be the ability to use current results to power more complex analyses of data, such as multivariate regression models to enable hypothesis testing.

Additionally, this survey included only brief responses about perceived barriers. Conducting one‐on‐one interviews with oncology teams could provide additional context on how barriers associated with OtoM could be addressed, as one‐on‐one interviews enable researchers to probe participants' perspectives to generate rich, nuanced information [70].

We have argued that overcoming the barriers to OtoM and building on the facilitators requires a programmatic approach that considers the perspectives of key stakeholders [23, 30]. By combining insights from oncology providers, audiologists, and patients, we aim to develop a proactive program of OtoM for VA that is streamlined and integrated into the cancer care continuum. Providing patient perspectives, although outside the scope of this report, is a limitation. A parallel survey of patients with cancer is currently underway to capture their perspectives on ototoxicity and on different strategies for addressing OtoM barriers, with the goal of ensuring that program design reflects patient preferences and priorities.

## 5 Conclusion

The results of this research demonstrate through provider surveys and VA‐wide administrative data that there is poor adherence to the recommended practice for managing ototoxicity in VA. Further, most surveyed VA oncology providers agreed on the importance of providing aspects of OtoM in many cancer care settings, particularly for patients treated with cisplatin. These findings suggest that audiological services should be included in multi‐disciplinary frameworks that aim to improve the Whole Health of patients with cancer.

Further research that includes specific OtoM strategies and utilizes patient‐level outcomes will be necessary to reveal how OtoM protocols can be optimized. Meanwhile, the OtoMIC survey results provided here identify contextually relevant barriers and facilitators for OtoM that can inform efforts to improve access to audiology care for patients with cancer to positively impact long‐term health outcomes for survivors.

## Author Contributions

All authors substantially contributed to the conception and design of the study, data analysis, data interpretation, and the drafting of the manuscript. All authors approved the final version of the manuscript. D.K.‐M. is responsible for the overall content of the manuscript.

## Funding

This material is the result of work by lead author (CL) that was initially funded by an NIH, NIDCD Ruth L. Kirschstein NRSA Short‐Term Institutional Research Training Grant (T35) [T35DC008764] to VA NCRAR, and was subsequently conducted as part of CL's Advanced Fellowship in Geriatrics, supported by the US Department of Veterans Affairs Office of Academic Affiliations, the Veterans Affairs Pittsburgh Health Care System, and the Department of Veterans Affairs Pittsburgh Geriatric Research, Education, and Clinical Center (GRECC). Work by other authors was supported in part by a VA RR&D Merit Review Award to Dawn Konrad‐Martin [#C3127zR/I01 RX003127] and Research Career Scientist Award [#RX22019] to Dawn Konrad‐Martin. This material is also the result of work supported with resources and the use of facilities at the VA Rehabilitation Research and Development (RR&D) National Center for Rehabilitative Auditory Research (NCRAR) [Center Award #C2361C/I50 RX002361] at the VA Portland Health Care System in Portland, Oregon.

## Ethics Statement

This study was performed in line with the principles of the Declaration of Helsinki. Approval was granted by the VA Portland Healthcare System/Oregon Health and Science University joint institutional review board, IRB (01/22/2020).

## Consent

The IRB granted a waiver of informed consent for this study because the survey participation was considered minimal risk and anonymous.

## Conflicts of Interest

Dawn Konrad‐Martin is listed as a coinventor on patents for a portable hearing test and testing device. The other authors declare no potential conflicts of interest.

## Supporting information


**Appendix S1:** cam471566‐sup‐0001‐AppendixS1.docx.

## Data Availability

The data that support the findings of this study are available on request from the corresponding author and with appropriate VA approvals. The data are not publicly available due to privacy or ethical restrictions.
