# Supplementary material for: Identifying Gaps and Opportunities to Improve Ototoxicity Management in Veterans With Cancer: Evidence From a Retrospective Cohort and Oncology Provider Survey
Source: Cancer Med. 2026 Feb 15;15(2):e71566. doi: 10.1002/cam4.71566 (PMC12906773; doi:10.1002/cam4.71566)
Supplement: Supplementary file 1 — Appendix S1: cam471566‐sup‐0001‐AppendixS1.docx. [file CAM4-15-e71566-s001.docx]

**Appendix A: Survey Background**

Table A1. Objectives of the Ototoxicity Management Interdisciplinary Communication (OtoMIC) Survey.

The objective of the OtoMIC survey is to solicit perspectives on the following topics: a) clinic, provider, and patient characteristics (e.g., years of practice, chief auditory complaint and age of the patient population); b) numbers of patients prescribed ototoxic medications at the respondent’s care setting; c) provider awareness of the prevalence of ototoxicity; d) provider awareness of symptoms of ototoxicity; e) provider awareness of patient risk factors for ototoxicity; f) the magnitude of the provider’s patient caseload with auditory or vestibular changes due to ototoxicity; g) who is responsible for specific elements of OtoM in the respondent’s care setting; h) the value of specific elements of an OtoM program; i) current ototoxicity care processes/prioritization of OtoM; j) how patients access OtoM services; k) perspectives on audiologists’ scope of practice; l) if the respondent’s institution follows an OtoM protocol; and m) if the respondent’s practice setting is open to updating OtoM processes and standard practices.

| Ototoxicity Management Interdisciplinary  Communication (OtoMIC) Survey – Oncology Version | | | | | |
| --- | --- | --- | --- | --- | --- |
| **CFIR Construct** | **Question** | **Objective** | | **Question Type** | |
| The purpose of this survey is to determine how best to provide hearing healthcare to patients receiving ototoxic drug therapies in the VA. Questions will ask about your experience with, knowledge of, and priorities regarding ototoxicity risk factors, symptoms, and management protocols. Instructions: Please answer each question by selecting the best answer. If you are unsure about how to answer a question, please give the best answer you can. | | | | | |
| **Inner setting** - Demographics  (Structural characteristics) | 1. *What is your primary medical specialty?*   - Nurse - Oncology Chief or Lead - Oncologist - Other (text box) | | a | | Multiple choice |
| **Demographics** | 2. *What is your terminal degree?*   - MD - PhD - MD/PhD - MS or MA - RN or LPN - Other (text box) | | a | | Multiple choice |
| **Demographics** | 3. *What is your VA practice setting?*   - VA Medical Center - Community Based Outpatient Clinic (CBOC) - Both | | a | | Multiple choice |
| **Demographics** | 4. *What is your practice location? (Please enter your VISN and 3 letter city abbreviation.)* | | a | | Open ended qualitative |
| **Demographics** | 5. *How many years have you been practicing in your field?*   - Less than a year - 1 to 3 years - 4 to 6 years - 7 to 10 years - 11 to 20 years - More than 20 years | | a | | Multiple choice |
| This section asks details about your patient population. If you are unsure of an answer, please choose the best/closest answer. | | | | | |
| **Outer setting** – Patient needs and resources  (Prevalence) | 6. *Approximately how many patients per month…*   - …do you treat with _____? (Oncologist) - …in your department are treated with _____? (Oncology Chief or Lead)   Rows   - Cisplatin - Carboplatin - Oxaliplatin - Radiation to the head   Columns   - Less than 5 - 5 to 10 - 11 to 15 - 16 to 20 - More than 20 - Unsure   6. *If _____ is part of the treatment, do oncology patients typically receive audiological monitoring for ototoxicity?* (Nurse; Oncologist; Oncology Chief or Lead)  Rows   - Cisplatin - Carboplatin - Oxaliplatin - Radiation to the head   Columns   - Yes - No - Unsure | | b | | Multiple selection (matrix) |
| **Outer setting** – Patient needs and resources  (Prevalence) | 7. *Approximately what percentage of your patients have had the following as a result of an ototoxic agent?*   - New or increased hearing loss - New or increased tinnitus - New or increased balance problems - Decreased quality of life as a result of one of the above - Unsure | | f | | Sliding scale percentage or select |
| **Individuals involved** – Evaluation of knowledge  (Prevalence) | 8. *What is the prevalence of ototoxic hearing loss among oncology patients receiving the following ototoxic agents?*   - Cisplatin - Carboplatin - Oxaliplatin - Radiation to the head - A combination of ototoxic agents - Unsure | | c | | Sliding scale percentage or select |
| This section of questions asks about your knowledge as a care provider about ototoxicity, including symptoms, risk factors, and monitoring practices. Please answer each question to the best of your ability. | | | | | |
| **Individuals involved** – Evaluation of knowledge | 9. *Which of the following are possible symptoms of ototoxicity?* ***(Select all that apply.)***  Rows   - Tinnitus - Hearing loss - Ear discharge - Indigestion/diarrhea - Dizziness - Lack of coordination - Fever - Bouncing or blurred vision - Tingling in extremities - Unsure - Other   Columns   - Yes - No - Unsure | | e | | Multiple selection (matrix) |
| **Individuals involved** – Evaluation of knowledge | 10. *Which of the following are known risk factors for ototoxicity?* ***(Select all that apply.)***   - Young age (Less than 5 years) - Older age - Inflammation - Hydration status - Dose and duration of ototoxic therapy - Renal insufficiency or insult - Hepatic failure - Metastasis - Good pre-treatment hearing - Poor pre-treatment hearing - Exposure to head and neck radiation - Concomitant intake of other ototoxic agents - Family history of ototoxicity - Poor diet/nutrition - Unsure - Other (text box) | | e | | Multiple selection |
| **Individuals involved** – Evaluation of knowledge | 11. *For the following scenario please select the answer(s) that best reflect your action plan.*   - A family member of a patient brings up that the patient has had a hard time following conversations in a noisy environment since their last cycle of cisplatin. - Refer to audiologist for hearing, tinnitus or balance management - Consider changing the dosage of cisplatin in the next cycle - Consider changing the agent in the next cycle - Provide counseling - Increase the frequency of ototoxicity monitoring - No treatment change - Other (text box) | | i, k | | Multiple selection |
| **Individuals involved** – Evaluation of knowledge | 12. *For the following scenario please select the answer(s) that best reflect your action plan.*   - A patient reports ringing in their ears before they are supposed to start a new cycle of carboplatin and radiation. - Refer to audiologist for hearing, tinnitus or balance management - Consider changing the dosage of carboplatin in the next cycle - Consider changing the agent in the next cycle - Provide counseling - Increase the frequency of ototoxicity monitoring - No treatment change - Other (text box) | | i, k | | Multiple selection |
| **Individuals involved** – Evaluation of knowledge | 13. *For the following scenario please select the answer(s) that best reflect your action plan.*   - The audiologist has confirmed that after receiving a cumulative dose of 170 mg/m2 of cisplatin a patient has had a significant hearing shift compared with their pre-treatment baseline evaluation. This patient will require a hearing aid. The patient is concerned about the persistent ringing and loss of hearing they have experienced since their last dose of cisplatin and is worried about progression of the hearing loss with further treatment. The tumor response to the treatment to the drug has been good. - Refer to audiologist for hearing, tinnitus or balance management - Consider changing the dosage of cisplatin in the next cycle - Consider changing the agent in the next cycle - Provide counseling - Increase the frequency of ototoxicity monitoring - No treatment change - Other (text box) | | i, k | | Multiple  selection |
| **Individuals involved** – Evaluation of knowledge | 14. *What do you believe is an appropriate schedule of ototoxicity monitoring tests for the results to be useful/actionable for you and your patients?* ***(Select all that apply.)***  *For clarification: "Treatment" refers to entire regimen; "cycle" refers to the repeated portion of the regimen.*  Rows   - Cisplatin - Carboplatin - Oxaliplatin - Radiation to the head   Columns   - No monitoring is needed if patient is counseled about risks - Beginning and end of treatment - Prior to administration of each dose - After every cycle - When patient reports ototoxic effects/symptoms - Unsure | | i | | Multiple selection (matrix) |
| **Individuals involved** – Evaluation of knowledge | 15. *Do you have a preferred ototoxicity monitoring schedule not reflected in the previous question?* | | i | | Open ended qualitative |
| **Individuals involved** – Evaluation of knowledge | 16. *Please rank the importance of the following factors for determining the appropriate schedule of ototoxicity monitoring tests (with 1 as the most important).*  Rows   - Cost - Feasibility (availability of patients, staff, and equipment) - Patient preference - Patient’s current health status - Relevance of test results to treatment plan - Relevance of test results to rehabilitation plan   Columns   - 1 - 2 - 3 - 4 - 5 - 6 | | i | | Ranking (1-6) |
| This section addresses current practices related to ototoxic symptoms including hearing loss, tinnitus, and vestibular effects. | | | | | |
| **Inner setting** - Network and communications  (Current methods/practice) | 17. *At your facility, which clinical team member is responsible for the following?* ***(Select all that apply.)***  Rows   - Inform patients of the risks for ototoxicity - Monitor patient reported symptoms - Monitor hearing during treatments with ototoxic agents - Counseling patients who develop hearing loss as a result of ototoxic agents - Providing patients with hearing aids and rehabilitation   Columns   - Audiologist - Oncology team - No specifically assigned provider - Unsure | | g | | Multiple selection (matrix) |
| **Inner setting** – Network and communications  (Current methods/practice) | 18. *For patients receiving a highly ototoxic agent (cisplatin or carboplatin),…*   - *…do you:* (Nurse; Oncologist) - …*does your service:* (Oncology Chief or Lead)   Rows   - Refer patients to audiology for baseline examination prior to starting oncology treatment? - Counsel patients about possible ototoxicity when using known ototoxic agents? - Refer patients to audiology for monitoring of ototoxic symptoms throughout treatment? - Refer patients to audiology for counseling? - Refer to audiology for follow-up examination after the conclusion of oncology treatment?   Columns   - Yes - Sometimes - No, but I’d like to - No, but it’s not necessary - Unsure | | g, i | | Multiple choice (matrix) |
| **Inner setting** –  Network and communications  (Current methods/practice) | 19. *How do patients at your facility access ototoxicity monitoring and/or management?* ***(Select all that apply.)***   - Referral from Pharmacy - Referral from Primary Care - Referral from Audiologist - Referral from Oncology Team - Self-referral - Unsure - Other (text box) | | j | | Multiple selection |
| **Inner setting** - Network and communications  (Evaluation of knowledge for oncologists) | 20. *Which of the following interventions falls under the audiologist’s scope of practice?* ***(Select all that apply.)***   - Modification of medication - Prescribing hearing aids and other assistive devices - Balance management - Tinnitus management - Education and counseling about risks and impacts of ototoxicity - Pre- and post-treatment hearing tests - Unsure - Other (text box) | | k  (g, i) | | Multiple selection |
| The last set of questions is intended to gather information on the climate for change regarding new or updated ototoxicity management protocols. For clarification: Monitoring refers to symptom surveillance; management refers to rehabilitation and/or treatment. | | | | | |
| **Inner setting** - Implementation climate  (Capacity for change) | 21. *Please rank the importance of managing the following treatment side effects.****(Click and drag each choice up or down to change the ranking where 1 is the most important.)***   - Hearing loss - Loss of taste - Nausea - Neuropathy - Sexual dysfunction - Sleep disturbance - Tinnitus - Vestibular (balance) dysfunction - Other | | i, h | | Ranking (1-8/9) |
| **Inner setting** – Implementation climate | 22. *What barriers exist to implementing ototoxicity monitoring/management at your facility?* | | h, m | | Open ended qualitative |
| **Inner setting** - Implementation climate | 23. *What changes and/or additional resources would increase or improve access to ototoxicity management at your facility?* | | h, m | | Open ended qualitative |
| **Inner setting** - Implementation climate | 24. *Which of those changes would receive strong leadership support and/or clinician support at your facility?* | | h, m | | Open ended qualitative |
| **Inner setting** - Implementation climate | 25. *If ototoxicity monitoring/management is available at your site, to what extent do you (would you) value the following?*  Rows   - Early detection of hearing loss, tinnitus, and balance problems - Ability for ototoxicity monitoring to influence the cancer treatment plan - Hearing health education and counseling resources to the patient/family - Management of hearing loss, tinnitus and/or balance issues **during** treatment with ototoxic agent - Management of hearing loss, tinnitus, and/or balance issues **after** treatment with ototoxic agent - Baseline evaluation prior to starting oncology treatment - Point-of-care ototoxicity screening - At-home ototoxicity screening   Columns   - Extremely useful - Very useful - Moderately useful - Slightly useful - Not at all useful | | h | | Multiple choice (matrix) |
| **Inner setting –** Implementation climate | 26. *Do you have any additional comments or recommendations related to ototoxicity management at your facility?* | | h | | Open ended qualitative |
| This survey is licensed but is freely available for most uses. If you would like to use this survey or any portions of it, please contact Dawn Konrad-Martin (dawn.martin@va.gov). | | | | | |

Figure A1. Relationship between project goals and the Consolidated Framework for Implementation Research (CFIR) constructs used to develop and interpret the Ototoxicity Management Interdisciplinary Communication (OtoMIC) survey.


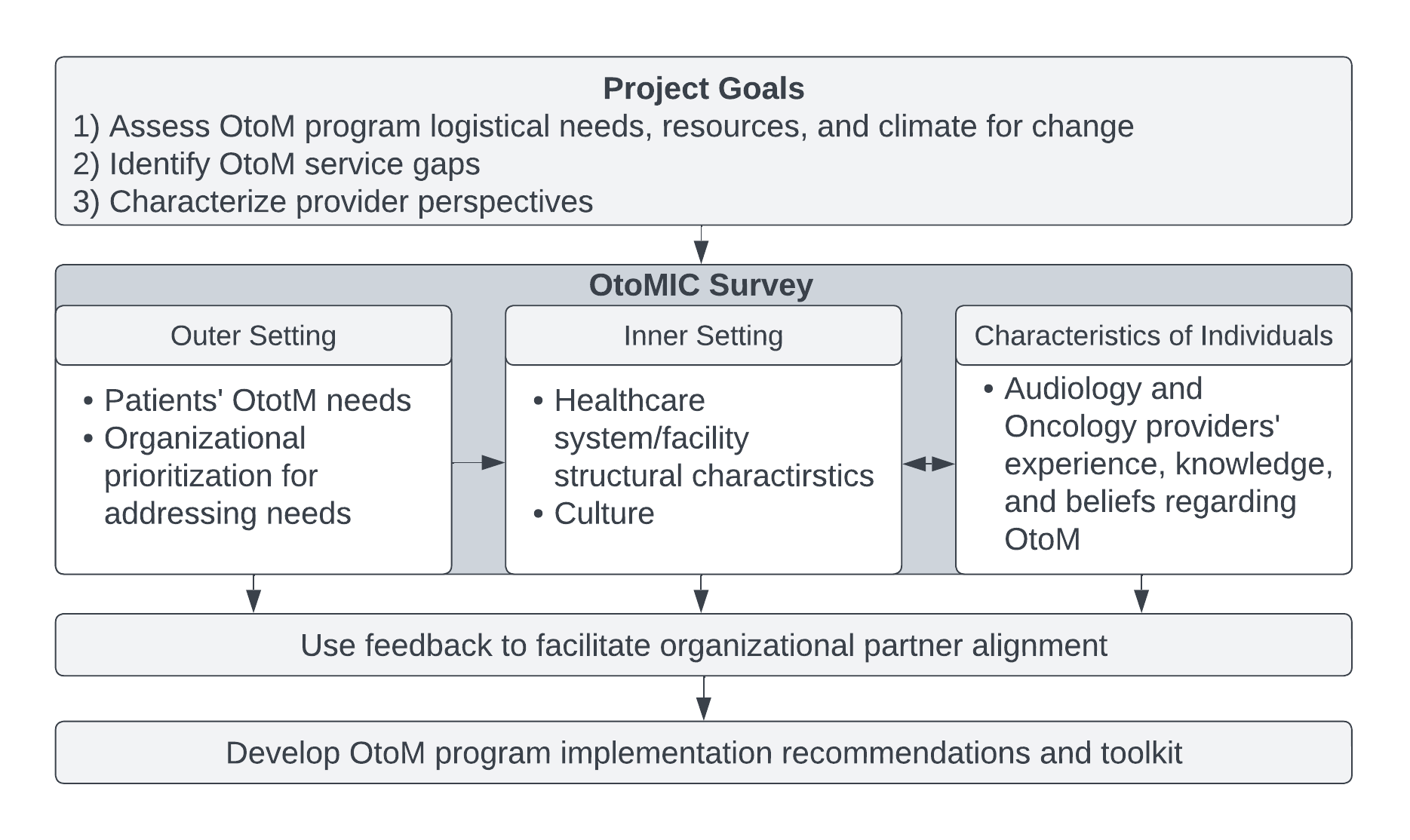


**Appendix B: Respondents’ Service Locations**

Figure B1. Map of the United States with Veterans Integrated Service Networks outlined and numbered. Stars indicate the service locations of respondents (total respondents, N=36; respondents with location information, N=33). Labels identify National Cancer Institute and Veterans Affairs Interagency Group to Accelerate Trials Enrollment sites.


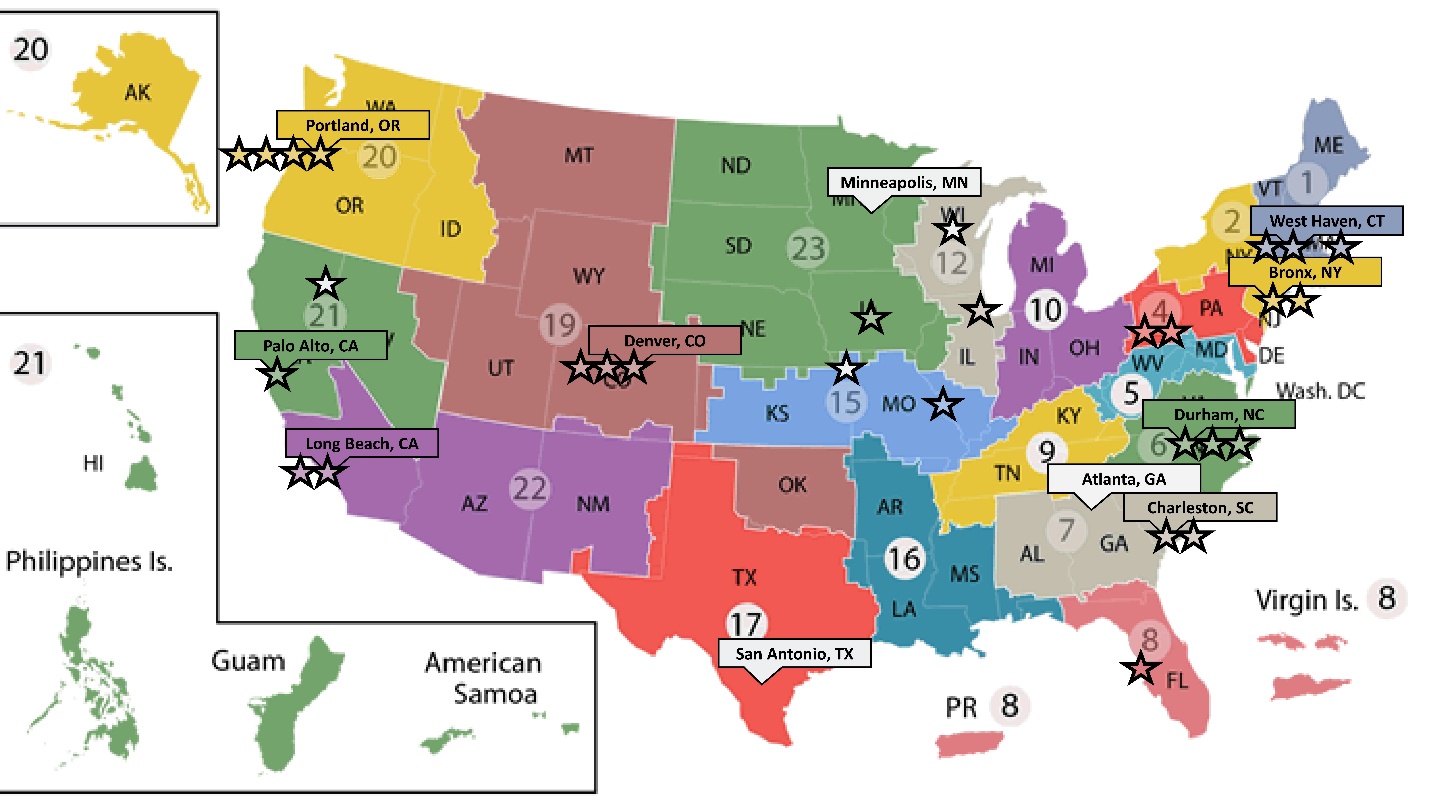


**Appendix C: Additional Results**

Figure C1. Ranking of ototoxicity management program considerations according to the importance of factors in determining a symptom surveillance schedule. A ranking of 1st indicates the most important and 6th the least important. (Question 16: Please rank the importance of the following factors for determining the appropriate schedule of ototoxicity monitoring tests.)

Figure C2. Perceived usefulness of various ototoxicity management program considerations. *One participant did not indicate the usefulness of two program considerations (N=29). (Question 25: If ototoxicity monitoring/management is available at your site, to what extent do you (would you) value the following?)

| Reported number of patients seen by oncology clinicians each month by ototoxic treatment type (N=8) | | | | |
| --- | --- | --- | --- | --- |
|  | **Fewer than 5 patients seen** | **5 to 10 patients seen** | **11 to 15 patients seen** | **16 to 20 patients seen** |
| **Cisplatin** | 5 oncology clinicians | 2 oncology clinicians | 0 oncology clinicians | 1 oncology clinician |
| **Carboplatin** | 3 oncology clinicians | 3 oncology clinicians | 2 oncology clinicians | 0 oncology clinicians |
| **Oxaliplatin*** | 4 oncology clinicians | 2 oncology clinicians | 1 oncology clinician | 0 oncology clinicians |
| **Radiation** | 5 oncology clinicians | 2 oncology clinicians | 0 oncology clinicians | 1 oncology clinician |

Table C1. Caseload of oncology patients per month by ototoxic treatment type.

Reported number of patients seen by oncology clinicians each month (columns) by ototoxic treatment type (rows). Many respondents chose not to answer this question. *One participant did not report the number of patients seen for oxaliplatin (N=7). (Question 6: Approximately how many patients per month do you treat with [drug]?)

Figure C3. Reported prevalence of side effects across respondents’ caseloads attributed to an ototoxic agent. (Question 7: Approximately what percentage of your patients have had the following as a result of an ototoxic agent?)


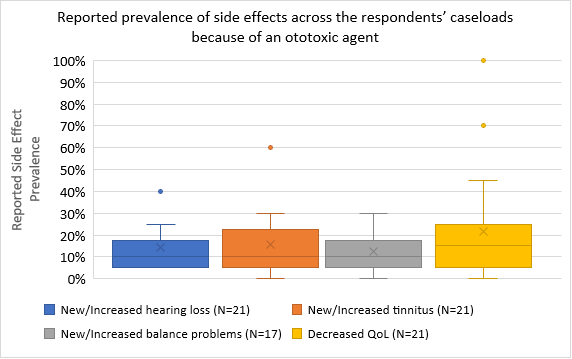


Figure C4. Estimated prevalence of ototoxic hearing loss by treatment type. Respondents’ estimates were lower than prevalence rates reported in the literature. (Question 8: What is the prevalence of ototoxic hearing loss among oncology patients receiving the following ototoxic agents?)


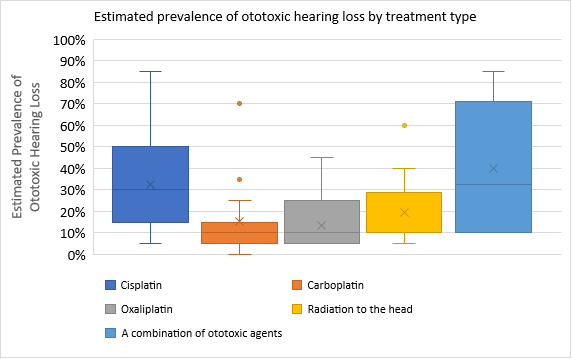


Table C2. Multidisciplinary team member responsibilities for ototoxicity management (OtoM).

Respondents selected all applicable provider options (columns) for each OtoM responsibility (rows). Numeric values and color gradations indicate the percentage of respondents who selected a given response; higher numbers and darker colors represent more common responses. (Question 17: At your facility, which clinical team member is responsible for the following?)

| **Multidisciplinary team member responsibilities for ototoxicity management** | | | | |
| --- | --- | --- | --- | --- |
|  | **Audiologist** | **Oncology team** | **No specifically assigned provider** | **Unsure** |
| **Inform patients of the risks for ototoxicity** (N=33) | 5 (**15%**) | 32 (**97%**) | 2 (**6%**) | 0 (**0%**) |
| **Monitor patient reported symptoms** (N=33) | 8 (**24%**) | 30 (**91%**) | 4 (**12%**) | 0 (**0%**) |
| **Monitor hearing during treatments with ototoxic agents** (N=33) | 18 (**55%**) | 24 (**73%**) | 4 (**12%**) | 1 (**3%**) |
| **Counsel patients who develop adverse symptoms as a result of ototoxic agents** (N=33) | 22 (**67%**) | 29 (**88%**) | 2 (**6%**) | 0 (**0%**) |
| **Provide patients with hearing aids and rehabilitation** (N=33) | 30 (**91%**) | 4 (**12%**) | 1 (**3%**) | 2 (**6%**) |

Figure C5. Oncology providers’ perceptions of which interventions fall within audiologists’ scope of practice. Respondents selected all applicable options. (Question 20: Which of the following interventions falls under the audiologist’s scope of practice?)

Table C3. Reported referral sources for ototoxicity monitoring and/or management.

Respondents selected all applicable referral sources. (Question 19: How do patients at your facility access ototoxicity monitoring and/or management?)

| **How do patients at your facility access ototoxicity monitoring and/or management (N=32)?** | |
| --- | --- |
| Referral from oncology team | 27 (**84**%) |
| Referral from primary care | 10 (**31%**) |
| Self-referral | 9 (**28%**) |
| Referral from audiologist | 7 (**22%**) |
| Unsure | 4 (**13%**) |
| Referral from pharmacy | 3 (**9%**) |
| Other | 1 (**3%**) |

Figure C6. Reported actions in response to patients receiving a highly ototoxic agent (cisplatin or carboplatin). Respondents could select multiple responses. (Question 18: For patients receiving a highly ototoxic agent, do you…)
